# Cause‐specific mortality after a diagnosis of ductal carcinoma in situ: Associations with screening and socio‐economic status

**DOI:** 10.1002/ijc.70112

**Published:** 2025-08-25

**Authors:** Renée S. J. M. Schmitz, Alexandra W. van den Belt‐Dusebout, Maartje van Seijen, Ellen A. J. Verschuur, Frederieke H. van Duijnhoven, Michael Schaapveld, Esther H. Lips, Jelle Wesseling, Marjanka K. Schmidt

**Affiliations:** ^1^ Division of Molecular Pathology Netherlands Cancer Institute Amsterdam The Netherlands; ^2^ Dutch Breast Cancer Society (‘Borstkanker Vereniging Nederland’) Utrecht The Netherlands; ^3^ Department of Surgery The Netherlands Cancer Institute – Antoni van Leeuwenhoek Amsterdam The Netherlands; ^4^ Division of Psychosocial Research and Epidemiology The Netherlands Cancer Institute Amsterdam The Netherlands; ^5^ Department of Pathology the Netherlands Cancer Institute – Antoni van Leeuwenhoek Amsterdam The Netherlands; ^6^ Department of Pathology Leiden University Medical Center Leiden The Netherlands; ^7^ Department of Clinical genetics Leiden University Medical Center Leiden The Netherlands

**Keywords:** breast cancer death, cause‐specific mortality, ductal carcinoma in situ (DCIS), screening, socio‐economic status (SES)

## Abstract

The lower all‐cause mortality in women with Ductal carcinoma in situ (DCIS) compared with the general population has been hypothesized to be due to a “healthy‐user effect,” but this has not been studied in large cohorts. In a population‐based, retrospective cohort study comprising 18,942 women with primary DCIS between 1999 and 2015 in the Netherlands, the cumulative incidence of breast cancer death (BCD) was estimated using death by other cause as a competing risk. The cause‐specific mortality risk of women with DCIS was compared with that of the Dutch female population. Multivariable competing risk regression was used to quantify the effects of the method of detection and socio‐economic status (SES). With 289 BCDs, the 10‐year cumulative incidence of BCD was 1.3% (95% CI, 1.1–1.5). Compared to the Dutch female population, women with DCIS had a 2.1‐times higher risk of BCD, but a 7% lower risk of all‐cause mortality. Women with screen‐detected DCIS had lower risks of BCD compared to women with non‐screen‐detected DCIS (subdistribution hazard ratio [sHR]:0.60, 95% CI 0.47–0.77), as did women with high SES versus low SES (sHR 0.54, 95% CI 0.30–0.97) in the first 4 years of follow‐up, adjusted for age and year at diagnosis, and DCIS characteristics. In conclusion, overall mortality in women with DCIS is not higher compared to the Dutch female population, though death due to invasive breast cancer is increased. Within all women with DCIS, those with screen‐detected DCIS or high SES had lower BCD and all‐cause mortality, suggesting a healthy‐user effect.

## Graphical abstract

What's new?

Ductal carcinoma in situ (DCIS) fails to progress to invasive breast cancer (IBC) in about four‐fifths of women. Nonetheless, once advanced to IBC, risk of death increases. Here, the authors investigated breast cancer mortality in Dutch women with DCIS in light of potential associations with socioeconomic status (SES) and DCIS detection method. Analyses show that women with screen‐detected DCIS and high SES have a lower risk of breast cancer death and all‐cause mortality, relative to the Dutch female population. The results suggest the existence of a “healthy‐user effect,” driven by screening attendance and SES, among Dutch women with DCIS.

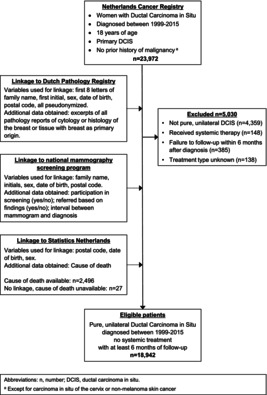

**Abbreviations**
AEM: absolute excess mortality
BCD: breast cancer death
BCS: breast conserving surgery
CBS: Statistics Netherlands
CI: confidence interval
DCIS: ductal carcinoma in situ
EBCTCG: Early Breast Cancer Trialists' Collaborative Group
ET: endocrine treatment
IBC: invasive breast cancer
mm: millimeter
MST: mastectomy
NCR: Netherlands Cancer Registry
p: p‐value
Palga: Dutch Nationwide Pathology Databank
RT: radiotherapy
SEER: Surveillance, Epidemiology, and End Results
SES: socio‐economic status
sHR: subdistribution hazard ratio
SMR: standardized mortality ratio
TNM: Tumor, Node, Metastasis
UK: United Kingdom

## 1 INTRODUCTION

Ductal carcinoma in situ (DCIS) is a non‐obligatory precursor to invasive breast cancer (IBC).^1^ In itself, DCIS is not lethal. However, DCIS may progress to IBC, potentially leading to an increased risk of dying from breast cancer. Importantly, previous studies have reported that up to 80% of DCIS lesions will never progress to IBC and the risk of subsequent IBC after treatment for DCIS is low.^2^, ^3^, ^4^, ^5^ However, there are no valid predictors that can, with sufficient accuracy, identify which DCIS lesions will progress to IBC. Therefore, DCIS is treated similarly to early‐stage IBC with surgery, either a mastectomy or breast conserving surgery, often followed by radiotherapy (RT), and in some countries endocrine treatment (ET). While reducing the risk of subsequent IBC, neither RT nor ET has been shown to decrease breast cancer death (BCD) in women with DCIS.^6^, ^7^, ^8^, ^9^ As such, there is a concern for overtreatment in women with DCIS.^1^, ^3^, ^10^, ^11^, ^12^

Thus, for DCIS management decisions and the potential de‐escalation of treatment, as well as for public health planning and policy making, it is important to accurately quantify the mortality rates of women with DCIS compared to women in the general population. Reported risk factors for BCD include younger age at diagnosis, higher DCIS grade, larger DCIS size, and non‐screen detected DCIS.^8^, ^9^, ^13^, ^14^, ^15^ A population‐based cohort from the United Kingdom comprising 35,024 women with screen detected DCIS reported a 1.7‐times higher risk of BCD compared to women in the general population (95% CI 1.52–1.90), whereas all‐cause mortality was lower in women with screen detected DCIS compared to the general population (observed: expected ratio 0.77, 95% CI 0.74 to 0.81).^8^ Studies from the United States, including data from the Surveillance, Epidemiology, and End Results (SEER) database reported 1.8‐ and 3.4‐times higher risk of BCD in women with DCIS compared to the general female population.^9^, ^16^ Similarly, in a previously published Dutch population‐based study, DCIS patients >50 years had a 2.8‐times higher risk of BCD, but a lower risk of death from all other causes compared with the general female population.^14^

It has been hypothesized that the lower all‐cause mortality in women with DCIS is due to a “healthy‐user effect” as the majority of DCIS lesions are diagnosed through screening and it has been widely reported that women who take part in population‐based screening programmes are of generally higher socio‐economic status (SES) and better overall health.^17^, ^18^, ^19^, ^20^ Furthermore, lower SES has been shown to be associated with a higher risk of diseases linked to lifestyle behaviors, such as cardiovascular disease, diabetes, and several types of cancer.^21^, ^22^, ^23^ To date, the association of SES and BCD after DCIS and the hypothesis of the healthy‐user effect in women with DCIS have not been studied in large cohorts.

Therefore, this study aimed to report accurate cause‐specific mortality after DCIS and to further investigate this hypothesized healthy‐user effect by analyzing the associations of SES and method of detection with cause‐specific mortality after treatment for DCIS.

## 2 MATERIALS AND METHODS

### 2.1 Data collection

Data from women diagnosed with DCIS were obtained from the Netherlands Cancer Registry (NCR)^24^ (Supplementary Figure S1), including data on patient‐, disease‐, and treatment‐characteristics and complete follow‐up on subsequent breast events and vital status until 01‐02‐2020. All primary diagnoses of DCIS and subsequent breast events or distant metastasis were verified using the pathology reports provided by the Dutch Nationwide Pathology Databank (Palga).^25^, ^26^ As the NCR registers primary diagnoses and not recurrences, subsequent invasive or contralateral in situ breast events were registered, but subsequent ipsilateral DCIS events were not and therefore extracted from Palga data. Information on the method of detection was provided by the NCR in collaboration with the breast cancer screening organisation and the cause of death by Statistics Netherlands (CBS). The general Dutch female population was used as a reference population for expected cause‐specific mortality. Sex‐, age‐, and calendar‐year specific mortality rates were obtained from CBS.^27^

### 2.2 Participants

The cohort initially comprised all 23,972 women diagnosed with primary DCIS between 1999 and 2015 (Supplementary Figure S1). Women with a non‐pure DCIS, a history of IBC or DCIS, missing information on treatment or follow‐up, follow‐up shorter than 6 months, or diagnosed with subsequent IBC within 6 months after the primary DCIS diagnosis were excluded. As systemic treatment, in any form, is not standard treatment for DCIS in the Netherlands and thus rarely prescribed, women who received systemic treatment for DCIS were excluded. In total, 5,030 women with DCIS were excluded, leaving 18,942 women with pure, primary DCIS for the analyses.

### 2.3 Definitions and data coding

Pure, primary DCIS was defined as no history of IBC or DCIS and no presence of (micro)invasive‐ or Paget's disease. Primary diagnosis and treatment were defined within 6 months prior to, or after first biopsy. Age at diagnosis was divided into six categories: <40, 40–49, 50–59, 60–69, 70–79, and >80. SES was scored from 1 to 10, based on the average income in the postal code zone at the time of diagnosis; these scores were categorized into low (0–3), medium (4–7) or high (8–10) SES.^28^ SES could not be determined for eight patients, for whom SES was scored as the median (6). Missing values in other variables were included in an unknown category. Screen‐detected DCIS was defined as a DCIS diagnosis within 2 years after a screening mammogram in the national biennial program for women aged 50–75, for which the woman was referred. DCIS size was divided into three categories, in line with TNM staging criteria for IBC: <20 mm, 20–49 mm, >49 mm.^29^ Margin status was defined according to current treatment guidelines^30^; clear margins: ≥2 mm and involved margins <2 mm, after final surgery. Cause of death was coded according to the ICD‐10^31^ and categorized as BCD, respiratory cancer death, non‐malignant circulatory, or respiratory death. For 27 patients who had died, linkage with Statistics Netherlands was unsuccessful; thus, cause of death was scored as unknown.

### 2.4 Statistical analyses

Time at risk started 6 months after the date of the primary DCIS diagnosis and ended at the date of death, or February 1st 2020. Cumulative incidence of BCD was estimated using death from other causes as a competing risk. Univariable and multivariable competing risk regression analyses were used to quantify the associations of method of detection and SES with BCD, estimating the subdistribution hazard ratios (sHR) and 95% confidence intervals (95% CI). Variables included in the multivariable model were SES at diagnosis, method of detection, year of diagnosis, age at diagnosis, DCIS size, DCIS grade, treatment type, and margin status. The proportional hazard assumption was violated for SES. Therefore, a split at the four‐year time point was added to the model.

Standardized mortality ratios (SMRs) were calculated as the ratio of the observed numbers of deaths in the study cohort and the sex‐, age‐, and calendar year‐specific expected cause‐specific mortality, taking into account the person‐years of observation in the cohort. Absolute excess mortality (AEM) was calculated by the observed number of deaths minus the number expected in the cohort, divided by the number of person‐years at risk in the cohort, multiplied by 10,000.^32^

All tests of statistical significance were two‐sided. A p‐value of <0.05 was considered statistically significant. All analyses were performed using STATA/SE 15.0 (StataCorp LP, College Station, TX).

## 3 RESULTS

For this study, 18,942 women diagnosed with pure, primary DCIS between 1999 and 2015 in the Netherlands were included. Patient‐ and disease characteristics are described in Table 1. Median follow‐up time was 10 years. Medium SES was most prevalent in the study (39%) and most DCIS lesions (64%) were screen‐detected. Roughly half of all women were treated with breast conserving surgery (BCS) and radiotherapy (RT), 11% with BCS only, and 38% with mastectomy (MST). Eleven percent of women had a subsequent breast event during follow‐up until 01‐02‐2020, and 2,523 women (13%) had died. Of those, 289 (11%) died from breast cancer, of whom 51% had a registered IBC or distant breast cancer metastasis prior to death; 555 (22%) died from circulatory diseases, and 168 died from respiratory diseases (7%).

**TABLE 1:** Patient characteristics at DCIS diagnosis.

	All patients *n* = 18,942	Women who died from breast cancer *n* = 289
Age at diagnosis in years (median, IQR)	58 (51–66)	59 (51–68)
Follow‐up time in years (median, IQR)	10 (7–14)	8 (5–12)

	*n* (%)	*n* (%)
Year of diagnosis		
1999–2005	5847 (31)	184 (64)
2006–2010	5427 (29)	72 (25)
2011–2015	7668 (40)	33 (11)
Socio‐economic status at year of diagnosis		
Low	5046 (27)	82 (45)
Medium	7478 (39)	117 (48)
High	6418 (34)	90 (6)
Method of detection		
Non‐screen‐detected	5935 (31)	130 (45)
Screen‐detected	12,129 (64)	140 (48)
Unknown	876 (5)	19 (7)
DCIS size		
<20 mm	6388 (34)	62 (21)
20–49 mm	3474 (18)	43 (15)
≥50 mm	1426 (8)	23 (8)
Unknown	7654 (40)	161 (56)
DCIS grade		
Grade 1	2831 (15)	23 (8)
Grade 2	5929 (31)	80 (28)
Grade 3	8934 (47)	155 (54)
Unknown	1248 (7)	31 (11)
Treatment		
Breast conserving surgery + radiotherapy	9643 (51)	119 (41)
Breast conserving surgery only	2130 (11)	45 (16)
Mastectomy	7169 (38)	125 (43)
Margin status		
Clear (≥2 mm)	13,583 (72)	160 (55)
Involved (<2 mm)	1596 (8)	29 (10)
Unknown	3763 (20)	100 (35)
Vital status		
Alive	16,427 (87)	N.A.
Deceased	2515 (13)	289 (100)

Abbreviations: DCIS, ductal carcinoma in situ; IQR, inter quartile range; mm, millimetre; *n*, number.

**FIGURE 1:**
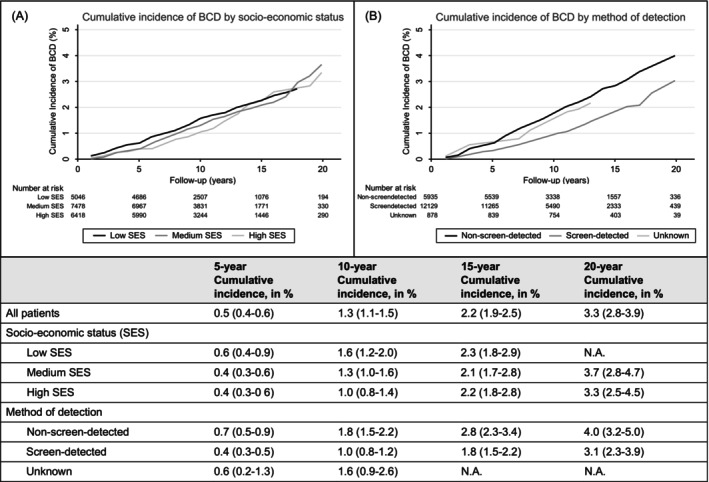
Cumulative incidences of breast cancer death by socio‐economic status and method of detection. Cumulative incidences were calculated with death by other causes as a competing event. (A) Cumulative incidences of breast cancer death by socio‐economic status; (B) Cumulative incidences of breast cancer death by method of detection. BCD, breast cancer death; SES, socio‐economic status; N.A., not applicable.

Cumulative incidences of BCD by SES and method of detection are shown in Figure 1. Overall, the cumulative incidences of BCD were low, with 0.5% (95% CI, 0.4–0.6) at 5 years, 1.3% (95% CI, 1.1–1.5) at 10 years, and 3.3% (95% CI, 2.8–3.9) at 20 years after DCIS diagnosis. The 5‐ and 10‐year cumulative incidences of BCD were 0.6% and 1.6% in women with low SES and 0.4% and 1.0% in women with high SES. Five‐ and 10‐year cumulative incidence of BCD were 0.4% and 1.0% in women with screen‐detected DCIS and 0.7% and 1.8% in women with non‐screen‐detected DCIS. Lastly, cumulative incidences of BCD were similar among treatment groups (Supplementary Figure S2).

In multivariable competing risk regression analyses, adjusted for method of detection, age at diagnosis, year of diagnosis, DCIS size, grade, treatment, and margin status (Table 2), high SES was associated with lower sHR for BCD, compared to low SES (sHR 0.54, 95% CI 0.30–0.97 *p* = 0.04) in the first 4 years of follow‐up. In contrast, after 4 years of follow‐up, high SES was associated with a higher sHR for BCD compared with low SES, although not statistically significant (sHR 1.92, 95% CI 0.97–3.80, *p* = 0.06). Women with screen‐detected DCIS had a lower sHR for BCD compared to women with non‐screen‐detected DCIS (0.60, 95% CI 0.47–0.77, *p* < 0.001). When compared with diagnosis between 1999 and 2005, sHRs for BCD for the more recent diagnosis periods were 0.63 (95% CI 0.47–0.85, *p* = 0.003) for 2006–2010, and 0.41 (95% CI 0.27–0.63, *p* < 0.001) for the diagnosis period 2011–2015. Risk of BCD significantly increased with age at diagnosis (sHR 1.01 per year, 95% CI 1.00–1.02, *p* = 0.02) and with higher DCIS grade (grade 2 sHR 1.91, 95% CI 1.19–3.06, *p* = 0.008; grade 3 sHR 2.41, 95% CI 1.53–3.78, *p* < 0.001) compared to DCIS grade 1. However, treatment type, margin status, or DCIS size were not associated with the risk of BCD.

**TABLE 2:** Competing risk regression analyses^a^ for breast cancer mortality after DCIS diagnosis.

	Follow‐up time	*n* BCD	Univariable sHR (95% CI)	*p*‐value	Multivariable sHR (95% CI)	*p*‐value
Socio‐economic status at diagnosis^b^	0.5–4 years					
Low		28	1.00 (ref)		1.00 (ref)	
Medium		25	0.60 (0.35–1.03)	0.064	0.60 (0.35–1.03)	0.065
High		19	0.53 (0.30–0.95)	0.034	0.54 (0.30–0.97)	0.040
Socio‐economic status at diagnosis^b^	>4 years					
Low		54	1.00 (ref)		1.00 (ref)	
Medium		92	1.87 (0.99–3.53)	0.053	1.88 (1.00–3.55)	0.051
High		71	1.95 (0.99–3.86)	0.054	1.92 (0.97–3.80)	0.060
Method of detection						
Non‐screen‐detected		130	1.00 (ref)		1.00 (ref)	
Screen‐detected		140	0.61 (0.48–0.77)	<0.001	0.60 (0.47–0.77)	<0.001
Unknown		19	0.73 (0.45–1.19)	0.212	0.52 (0.31–0.85)	0.010
Age at diagnosis^c^		289	1.01 (0.99–1.02)	0.316	1.01 (1.00–1.02)	0.020
Year of diagnosis						
1999–2005		184	1.00 (ref)		1.00 (ref)	
2006–2010		72	0.63 (0.48–0.83)	0.001	0.63 (0.47–0.85)	0.003
2011–2015		33	0.39 (0.26–0.58)	<0.001	0.41 (0.27–0.63)	<0.001
DCIS size						
<20mm		62	1.00 (ref)		1.00 (ref)	
20–49 mm		43	1.27 (0.86–1.87)	0.232	1.16 (0.77–1.74)	0.472
≥50 mm		23	1.74 (1.08–2.80)	0.023	1.59 (0.96–2.64)	0.073
Unknown		161	1.27 (0.86–1.87)	0.006	1.30 (0.92–1.82)	0.133
DCIS grade						
Grade 1		23	1.00 (ref)		1.00 (ref)	
Grade 2		80	1.73 (1.09–2.75)	0.020	1.91 (1.19–3.06)	0.008
Grade 3		155	2.14 (1.38–3.32)	0.001	2.41 (1.53–3.78)	<0.001
Unknown		31	2.01 (1.17–3.45)	0.011	1.72 (1.00–2.96)	0.052
Treatment						
Breast conserving surgery + radiotherapy		119	1.00 (ref)		1.00 (ref)	
Breast conserving surgery only		45	1.32 (0.94–1.88)	0.112	1.25 (0.86–1.82)	0.232
Mastectomy		125	1.22 (0.94–1.57)	0.130	0.94 (0.71–1.24)	0.648
Margin status						
Clear ≥2 mm		160	1.00 (ref)		1.00 (ref)	
Involved <2 mm		29	1.18 (0.79–1.75)	0.422	0.99 (0.66–1.50)	0.976
Unknown		100	1.30 (1.01–1.69)	0.045	0.93 (0.68–1.28)	0.665

Abbreviations: BCD, breast cancer death; CI, confidence interval; mm, millimeter; *n*, number; sHR, subdistribution hazard ratio.

^a^ Competing risk regression with death by other causes as a competing risk.

^b^ Proportional hazard assumption for socio‐economic status was violated; as such, follow‐up time for this variable was split for these analyses.

^c^ Per year.

Compared to the general Dutch female population, women with DCIS have a lower risk of death from any cause (SMR = 0.93; 95% CI, 0.89–0.96), absolute excess mortality (AEM) = −10.54 per 10,000 person‐years, and especially of circulatory death (SMR = 0.82; 95% CI 0.75–0.89) and of respiratory death (SMR = 0.72; 95% CI, 0.61–0.83) (Table 3). Women with high SES had significantly lower risks of dying from all causes, circulatory causes, and respiratory causes compared to the general population, whereas in women with low SES this was not observed. Similarly, women with screen‐detected DCIS had a lower risk of death from all causes, circulatory causes, and respiratory causes compared to the general population, while in women with non‐screen‐detected DCIS, only a decreased risk in circulatory causes was observed (Table 3). Women with DCIS had a 2.07‐times increased risk of dying from breast cancer (95% CI, 1.84–2.33; AEM = 7.94), compared to the general population (Table 4). This elevated risk was most pronounced in women with non‐screen‐detected DCIS (SMR, 3.33; 95% CI, 2.78–3.95) and less in women with screen‐detected DCIS (SMR, 1.57; 95% CI, 1.32–1.85) (Table 4). The risk of BCD compared to the general population did not differ significantly between women with low and high SES (low SES: SMR, 2.19; 95% CI, 1.74–2.72; high SES SMR, 1.96; 95% CI, 1.58–2.41). Women with high SES did have a lower risk of dying from respiratory cancers compared to the general population (SMR 0.65; 95% CI, 0.48–0.86), which was not observed in women with low SES.

**TABLE 3:** Cause‐specific standardized mortality ratios.

	All Causes				Circulatory diseases (non‐malignant)				Respiratory diseases (non‐malignant)			
	*n*	SMR^a^ (95% CI)	*p*‐value	AEM^b^	*n*	SMR^a^ (95% CI)	*p*‐value	AEM^b^	*n*	SMR^a^ (95% CI)	*p*‐value	AEM^b^
Overall	2515	0.93 (0.89–0.96)	<0.001	−10.54	555	0.82 (0.75–0.89)	<0.001	−6.42	168	0.72 (0.61–0.83)	<0.001	−3.54
Socio‐economic status at diagnosis												
Low	801	1.06 (0.99–1.14)	0.108	9.12	180	0.94 (0.81–1.09)	0.418	−2.39	67	1.02 (0.79–1.29)	0.921	0.23
Medium	1014	0.93 (0.87–0.99)	0.016	−10.62	231	0.85 (0.74–0.97)	0.011	−5.52	52	0.55 (0.41–0.72)	<0.001	−5.69
High	700	0.81 (0.75–0.87)	<0.001	−25.57	144	0.68 (0.57–0.80)	<0.001	−10.59	49	0.66 (0.49–0.87)	0.002	−3.93
Method of detection												
Non‐screen‐detected	871	1.01 (0.94–1.08)	0.878	0.78	184	0.77 (0.66–0.89)	<0.001	−0.83	26	0.79 (0.60–1.02)	0.069	−2.55
Screen‐detected	1363	0.89 (0.84–0.93)	<0.001	−15.54	291	0.84 (0.75–0.94)	0.002	−4.9	59	0.64 (0.51–0.79)	<0.001	−4.15
Unknown	281	0.92 (0.81–1.03)	0.155	−22.08	80	0.89 (0.71–1.11)	0.327	−8.59	85	0.88 (0.51–0.79)	0.61	−2.88
Age in years												
<40	29	3.41 (2.28–4.89)	<0.001	26.75	<10^c^	0.95 (0.02–5.30)	0.72	−0.67	<10^c^	0.00 (0.00–12.88)	0.751	−0.37
40–49	110	1.42 (1.17–1.71)	<0.001	12.62	<10^c^	0.91 (0.42–1.73)	0.95	−0.33	<10^c^	0.46 (0.06–1.67)	0.385	−0.91
50–59	447	0.94 (0.85–1.03)	0.18	−3.93	45	0.64 (0.47–0.86)	0.002	−3.36	19	0.54 (0.32–0.84)	0.004	−2.17
60–69	744	0.85 (0.79–0.91)	<0.001	−24.62	140	0.71 (0.60–0.84)	<0.001	−10.41	55	0.71 (0.53–0.92)	0.009	−4.2
70–79	991	0.94 (0.88–1.00)	0.036	−28.45	291	0.90 (0.80–1.01)	0.083	−13.06	72	0.74 (0.58–0.94)	0.01	−10.32
≥80	194	0.90 (0.78–1.04)	0.16	−120.59	69	0.91 (0.70–1.15)	0.442	−41.9	20	0.98 (0.60–1.52)	0.53	−1.86
Year of diagnosis												
1999–2005	1574	0.98 (0.94–1.03)	0.542	−2.87	367	0.84 (0.75–0.93)	0.001	−8.18	111	0.79 (0.65–0.96)	0.013	−3.33
2006–2010	586	0.86 (0.79–0.94)	<0.001	−16.73	119	0.78 (0.64–0.93)	0.005	−6.08	29	0.50 (0.34–0.72)	<0.001	−5.15
2011–2015	355	0.82 (0.73–0.91)	<0.001	−17.66	69	0.82 (0.64–1.03)	0.095	−3.45	28	0.76 (0.51–1.10)	0.163	−1.95

Abbreviations: AEM, absolute excess mortality; CI, confidence interval; *n*, number; SMR, standardized mortality ratio.

^a^ SMRs were calculated by determining the ratio of the observed cause‐specific mortality rates in the study cohort and the expected cause‐specific mortality rates in the general Dutch female population, taking into account the person‐years of observation in the cohort by sex, age, and year at DCIS diagnosis, and follow‐up interval.

^b^ AEM was calculated by the observed number of deaths in the cohort minus the expected in the general Dutch female population, divided by the number of person‐years at risk in the cohort, and multiplied by 10,000.

^c^ Due to privacy considerations, <10 was used in case of fewer than 10 women in the group.

**TABLE 4:** Cancer‐specific standardized mortality ratios.

	All malignancies				Breast cancer				Malignancies of the respiratory system			
	*n*	SMR^a^ (95% CI)	*p*‐value	AEM^b^	*n*	SMR^a^ (95% CI)	*p*‐value	AEM^b^	*n*	SMR^a^ (95% CI)	*p*‐value	AEM^b^
Overall	1030	1.11 (1.04–1.18)	0.001	5.27	289	2.07 (1.84–2.33)	<0.001	7.94	208	0.94 (0.81–1.07)	0.363	−0.75
Socio‐economic status at diagnosis												
Low	305	1.21 (1.08–1.35)	0.001	10.68	82	2.19 (1.74–2.72)	<0.001	9.05	72	1.21 (0.95–1.52)	0.130	2.52
Medium	432	1.15 (1.04–1.26)	0.005	7.44	117	2.08 (1.72–2.50)	<0.001	8.11	89	0.99 (0.80–1.22)	0.500	−0.09
High	293	0.97 (0.86–1.09)	0.625	−1.43	90	1.96 (1.58–2.41)	<0.001	6.9	47	0.65 (0.48–0.86)	0.002	−4.04
Method of detection												
Non‐screen‐detected	344	1.41 (1.27–1.57)	<0.001	16.15	130	3.33 (2.78–3.95)	<0.001	14.6	57	1.03 (0.78–1.33)	0.877	0.24
Screen‐detected	598	0.98 (0.91–1.07)	0.689	−0.92	140	1.57 (1.32–1.85)	<0.001	4.44	132	0.88 (0.74–1.04)	0.148	−1.57
Unknown	88	1.12 (0.90–1.38)	0.328	8.04	19	1.69 (1.02–2.64)	0.043	6.79	19	1.15 (0.69–1.79)	0.610	2.14
Age in years												
<40	23	5.12 (3.24–7.68)	<0.001	24.16	‐^c^	15.61 (8.78–22‐82)	<0.001	23.11	<10^c^	1.16 (0.03–6.44)	0.579	0.18
40–49	75	1.68 (1.32–2.10)	<0.001	11.78	33	3.26 (2.25–4.58)	<0.001	8.92	18	1.45 (0.86–2.30)	0.159	2.18
50–59	308	1.17 (1.04–1.31)	0.007	5.97	91	1.99 (1.61–2.45)	<0.001	6.02	78	1.04 (0.82–1.30)	0.741	0.43
60–69	349	0.98 (0.88–1.09)	0.756	−1.21	86	1.78 (1.42–2.19)	<0.001	6.98	69	0.80 (0.62–1.02)	0.067	−3.17
70–79	256	1.06 (0.93–1.20)	0.365	6.08	55	1.77 (1.34–2.31)	<0.001	10	41	0.92 (0.66–1.24)	0.632	−1.6
≥80	19	0.88 (0.53–1.38)	0.676	−14.85	<10^c^	1.70 (0.55–3.96)	0.351	11.84	<10^c^	0.32 (0.01–1.79)	0.364	−12.21
Year of diagnosis												
1999–2005	593	1.23 (1.13–1.33)	<0.001	12.56	184	2.53 (2.17–2.92)	<0.001	12.76	106	0.99 (0.81–1.20)	0.950	−0.15
2006–2010	269	1.03 (0.91–1.16)	0.631	1.45	72	1.85 (1.45–2.33)	<0.001	5.89	62	0.95 (0.73–1.21)	0.726	−0.62
2011–2015	168	0.90 (0.77–1.05)	0.189	−4.06	33	1.19 (0.82–1.67)	0.359	1.18	40	0.81 (0.58–1.11)	0.206	−2.06

Abbreviations: AEM, absolute excess mortality; CI, confidence interval; *n*, number; SMR, standardized mortality ratio.

^a^ SMRs were calculated by determining the ratio of the observed cause‐specific mortality rates in the study cohort and the expected cause‐specific mortality rates in the general Dutch female population, taking into account the person‐years of observation in the cohort by sex, age, and year at DCIS diagnosis, and follow‐up interval.

^b^ AEM was calculated by the observed number of deaths in the cohort minus the expected in the general Dutch female population, divided by the number of person‐years at risk in the cohort, and multiplied by 10,000.

^c^ Due to privacy considerations, <10 was used in the case of fewer than 10 women in the group. In the case of only one group smaller than 10, the smallest number following was omitted in order to prevent revealing the number below 10.

## 4 DISCUSSION

In our large, population‐based cohort, the absolute risk of BCD after treatment for DCIS was low (1.3% at 10 years and 3.3% at 20 years). Women with screen‐detected DCIS had lower risks of BCD compared to women with non‐screen‐detected DCIS, as did women with higher SES compared to women with low SES (in the first years of follow‐up). Compared to the general Dutch female population, women treated for DCIS had a 2.1‐times higher risk of BCD. In contrast, women with DCIS had a lower risk of dying from all causes, circulatory causes, and respiratory causes, and of cancer originated from the respiratory system. However, these decreased risks were only observed in women with medium and high SES, not in women with low SES. Similarly, these decreased risks were only observed in women with screen‐detected DCIS, not in women with non‐screen‐detected DCIS, which could support the hypothesis of a “healthy‐user effect” in women participating in the screening program.

The 20‐year cumulative incidence of BCD (3.3%) in our study is similar to those reported in a UK‐based screening cohort comprising 35,024 DCIS patients (3.8%) and two US‐based SEER cohorts of 144,524 and 108,196 DCIS patients (3.3%), published in 2015 and 2020.^8^, ^9^, ^16^ However, the 10‐year cumulative incidence of BCD (1.3%) in our cohort was lower compared to a SEER cohort study from 2022 of 29,515 patients (2.2%).^13^ Although the long‐term absolute risk of BCD was low, the risk of BCD was 2.1 times increased compared to the general Dutch female population, which is in line with the UK study (SMR 1.7) and the SMR of 1.8 in the SEER cohort study from 2015, but lower than the SMR of 3.1 in a SEER cohort study from 2020.^8^, ^9^, ^16^ However, this difference is likely to be due to a different methodology used in the 2020 SEER study: the SMR of 3.1 was calculated by comparing an observed cohort to a comparison cohort restricted to women who were cancer‐free at the age of diagnosis for women in the observed cohort.^16^ Whereas in our study, the entire Dutch female population was used as the comparison cohort. As such, the results of the two studies are difficult to compare. In both the UK study (SMR 1.7) and the SEER cohort study from 2015 (SMR 1.8), mortality rates in the general population were also used as reference rates, which may explain why these SMRs are more comparable to those found in our study.^8^, ^9^

Furthermore, in line with the SEER‐cohort study of 2022, our study confirms that factors associated with subsequent IBC differ from factors associated with BCD.^13^ Neither treatment type, nor margin status and DCIS‐size were significantly associated with the risk of BCD, whereas these are commonly reported risk factors for IBC.^33^ Other factors, such as a genetic component, lifestyle factors, or treatment of the subsequent breast cancer may be risk factors for BCD after DCIS, especially because the risk of death from all causes and BCD were reduced for higher SES and screen‐detected DCIS.

In this study, the addition of RT to BCS was not associated with a significantly lower BCD. This is in contrast with the SEER‐cohort studies, but is in line with the UK‐cohort and the prospective Early Breast Cancer Trialists' Collaborative Group (EBCTCG) trials. This EBCTCG meta‐analysis on RT benefit in DCIS reported a 10‐year cumulative risk of BCD of 4.1% for women receiving BCS + RT compared to 3.7% in women receiving BCS only.^6^, ^8^, ^9^, ^13^, ^16^, ^34^

Our study has strengths and some limitations. It is a large, contemporary population‐based study on cause‐specific mortality after DCIS, with detailed, mostly patient‐level information on patient‐, disease‐, and treatment‐characteristics and long‐term follow‐up. Record linkages between the nationwide cancer registry, pathology database, screening organization, and cause of death registry facilitated a wide variety of data regarding potentially important risk factors for cause‐specific mortality. Consequently, this study is the first, to our knowledge, considering data on SES and method of detection in analyzing the risk of BCD after DCIS.

Large data linkages are also affected by some technical challenges. The most notable is that half of all patients with BCD (49%) did not have an IBC diagnosis after their primary DCIS, which, although comparable to other studies,^9^, ^14^ seems a high percentage as we assume that DCIS in itself cannot metastasize. One explanation might be the imperfections in the registration of subsequent IBCs and causes of death, and linkages between different registrations. However, data checks for inconsistencies between the data provided by the NCR and CBS yielded no discrepancies. Since Dutch legislation prohibits CBS from exporting individual causes of death, it was impossible to directly compare the 140 discrepant breast cancer incidences and BCDs. However, using only the study ID numbers of a random sample of 40 deceased women from CBS, using NCR and Palga data revealed that the discrepancy observed was mostly explained by an IBC developed outside the available NCR follow‐up time or metastases diagnosed during DCIS follow‐up that turned out to originate from IBC. Also, the numbers of non‐breast malignancies provided by the NCR and CBS were equal. Therefore, mismatches or registration imperfections could not have largely influenced the results of this study.

Furthermore, although valuable data, the estimation of SES based on the average income of the postal code region of the address at the time of DCIS diagnosis instead of individual information is a limitation of the study. SES may vary within a postal code area, and SES does not need to be constant over a person's lifetime. However, the associations of high SES and health benefits observed in this study are in line with previous studies on the association between SES and overall health.^21^, ^22^, ^23^

The results of our study support the previously hypothesized healthy‐user effect in women who participate in breast cancer screening.^14^ It could be hypothesized that the elevated risk of BCD in the short term (first 4 years of follow‐up) in DCIS patients with lower SES is due to decreased access to healthcare. However, in the Netherlands, universal healthcare is available and health insurance is mandatory for all citizens.^35^ As such, no financial barriers for access to healthcare should exist, as also shown by a previous Dutch population‐based study reporting that SES did not affect the type of treatment given for stage I and II IBC.^36^ In the longer term (after 4 years of follow‐up), it is possible that part of the increased BCD risk in DCIS patients is also due to a healthy‐user effect. On average, women that are generally healthier are less likely to die from competing events, and thus live longer. As the risk of IBC increases with age, these women will eventually have a higher risk of BCD. Therefore, general health and SES have to be taken into account when interpreting the risk of BCD after DCIS.

This study confirms that, although women with DCIS have an increased risk of BCD compared to the general Dutch female population, absolute risks are low. Additionally, women diagnosed with DCIS, especially through population‐based screening, have decreased risks of dying from other causes, which could be due to a healthy‐user effect among women with screen‐detected DCIS.

## AUTHOR CONTRIBUTIONS

**Renée S. J. M. Schmitz:** Writing – original draft; writing – review and editing; investigation; visualization; formal analysis; data curation; conceptualization; methodology. **Alexandra W. van den Belt‐Dusebout:** Investigation; methodology; writing – original draft; writing – review and editing; data curation; conceptualization; formal analysis; supervision. **Maartje van Seijen:** Writing – review and editing; conceptualization; methodology; formal analysis. **Ellen A. J. Verschuur:** Writing – review and editing; conceptualization. **Frederieke H. van Duijnhoven:** Writing – review and editing. **Michael Schaapveld:** Methodology; conceptualization; writing – review and editing; software. **Esther H. Lips:** Conceptualization; writing – original draft; writing – review and editing; supervision; funding acquisition; methodology. **Jelle Wesseling:** Conceptualization; supervision; writing – review and editing; writing – original draft; funding acquisition; methodology. **Marjanka K. Schmidt:** Conceptualization; supervision; methodology; writing – original draft; writing – review and editing; funding acquisition.

## FUNDING INFORMATION

The PRECISION project was supported by Cancer Research UK and by KWF Kankerbestrijding (ref. C38317/A24043). Research at the Netherlands Cancer Institute is supported by Institutional grants of the Dutch Cancer Society (KWF Kankerbestrijding) and of the Dutch Ministry of Health, Welfare and Sport.

## CONFLICT OF INTEREST STATEMENT

All authors declare no financial relationships with any organizations that might have an interest in the submitted work in the previous 3 years; no other relationships or activities could appear to have influenced the submitted work.

## ETHICS STATEMENT

The data collection for the Dutch cohort was reviewed and approved by the NCR and Palga review boards (data study numbers NCR: K12.281, K17.321; data study numbers Palga: lzv990, lzv2017‐173). Data protection and curation was done in compliance with current ethical and data protection regulations.

## Supporting information


**Figure S1.** Flow chat.
**Figure S2.** Cumulative incidences of breast cancer specific death by treatment type. BCD, breast cancer death; BCS, breast conserving surgery; RT, radiotherapy; MST, mastectomy.

## Data Availability

The data sets generated and/or analyzed during the current study are not publicly available, as the study has used external data from the NCR, Palga, and Statistics Netherlands. To inquire for data access from the NCR please visit https://www.iknl.nl/en/ncr/apply-for-data (data request study numbers K12.281, K17.321). To inquire for data access from Palga, please contact aanvraag@palga.nl (data request numbers lzv990, lzv2017‐173). The non‐public cause of death microdata is accessible for statistical and scientific research from Statistics Netherlands, under certain conditions: For further information please contact microdata@cbs.nl. (Project number 9103). Further information is available from the corresponding author upon request.
