# Supplementary material for: Cause‐specific mortality after a diagnosis of ductal carcinoma in situ: Associations with screening and socio‐economic status
Source: Int J Cancer. 2025 Aug 25;158(4):873–83. doi: 10.1002/ijc.70112 (PMC12712357; doi:10.1002/ijc.70112)
Supplement: Supplementary file 1 — Figure S1. Flow chat. Figure S2. Cumulative incidences of breast cancer specific death by treatment type. BCD, breast cancer death; BCS, breast conserving surgery; RT, radiotherapy; MST, mastectomy. [file IJC-158-873-s001.pdf]

Cause-specific mortality after a diagnosis of ductal carcinoma in situ: associations with screening and socio-economic status

Renée S.J.M. Schmitz, Alexandra W. van den Belt-Dusebout, Maartje van Seijen PhD, Ellen A.J. Verschuur, Frederieke H. van Duijnhoven, Michael Schaapveld, Esther H. Lips, Jelle Wesseling, Marjanka K. Schmidt

Table of contents

- Supplementary Figure S1 p.2

- Supplementary Figure S2 p.3

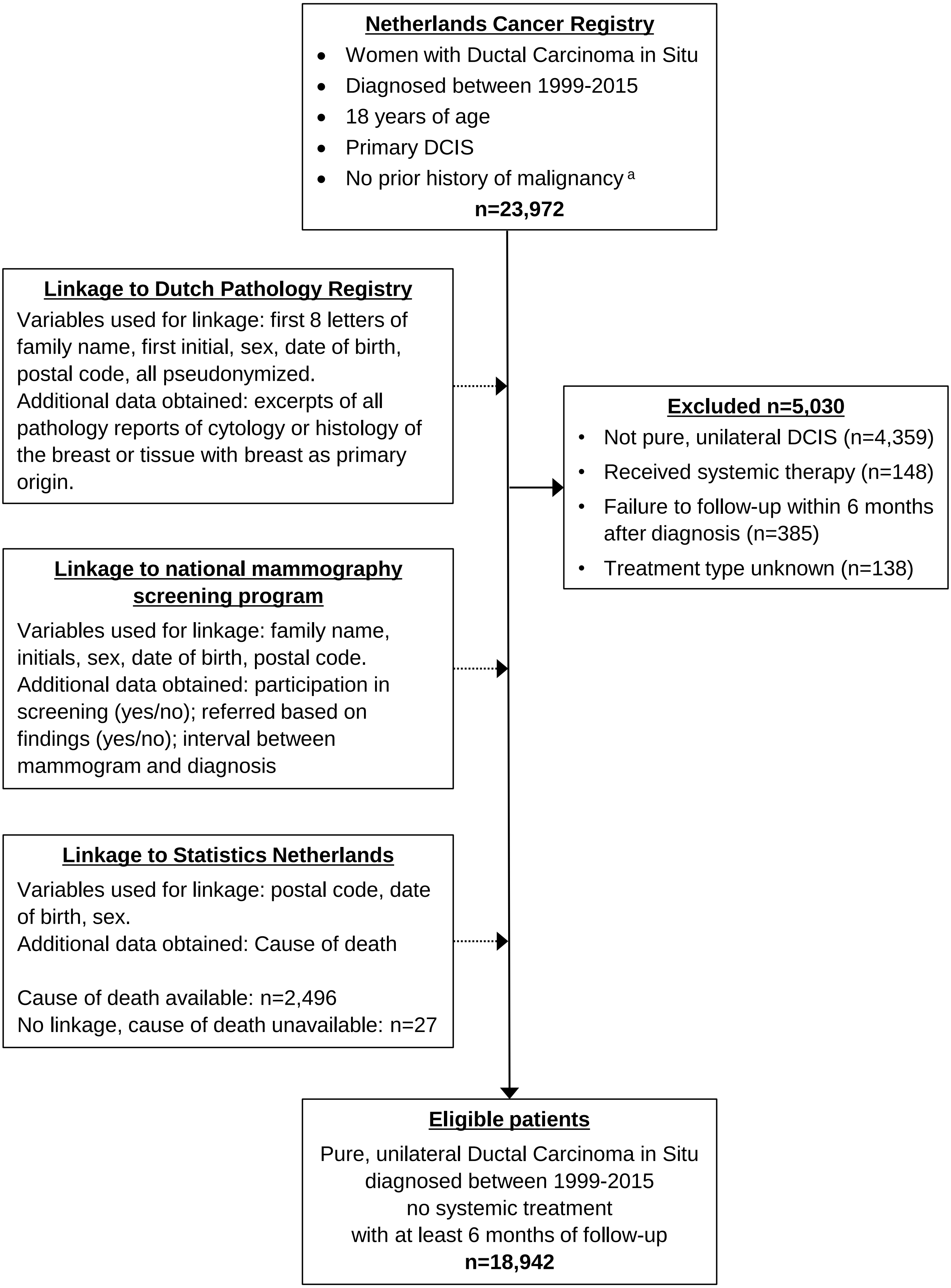

Supplementary Figure S1: Flowchart.

Abbreviations: n, number; DCIS, ductal carcinoma in situ.

[a] Except for carcinoma in situ of the cervix or non-melanoma skin cancer

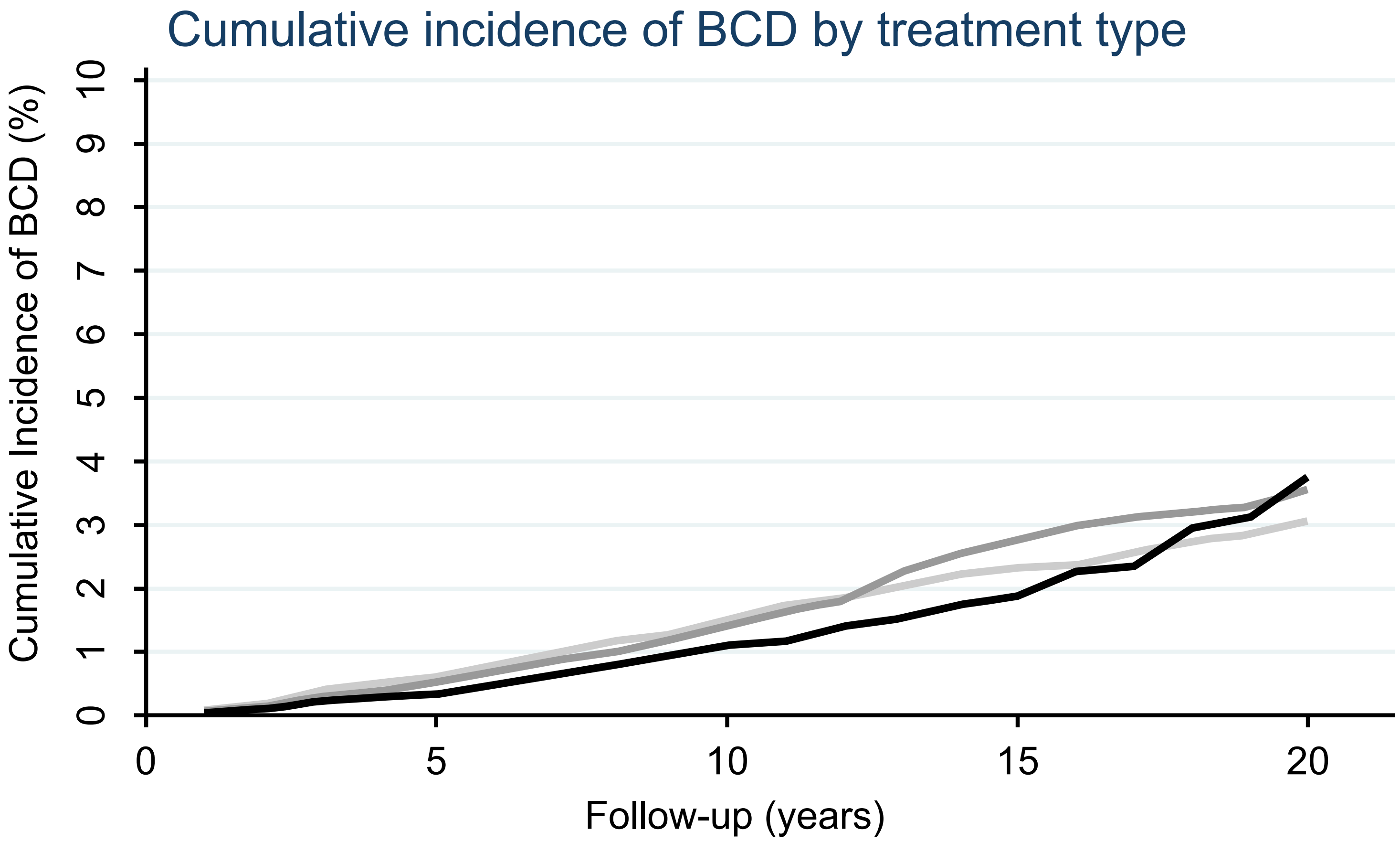

Number at risk

BCS+RT	9643	9025	4402	1692	232
BCS only	2130	1943	1212	724	237
MST	7169	6675	3968	1877	345

BCS+RT BCS only MST

	5-year Cumulative incidence, in %	10-year Cumulative incidence, in %	15-year Cumulative incidence, in %	20-year Cumulative incidence, in %
BCS+RT	0.3 (0.2-0.5)	1.1 (0.9-1.4)	1.9 (1.5-2.3)	3.8 (2.7-5.1)
BCS only	0.5 (0.3-0.9)	1.4 (0.9-2.0)	2.8 (2.0-3.7)	3.6 (2.5-4.8)
Mastectomy	0.6 (0.4-0.8)	1.5 (1.2-1.8)	2.3 (1.9-2.8)	3.1 (2.4-3.9)

Supplementary Figure S2: Cumulative incidences of breast cancer specific death by treatment type.

Abbreviations: BCD, breast cancer death; BCS, breast conserving surgery; RT, radiotherapy; MST, mastectomy. Cumulative incidences were calculated using death by other cause as competing event.
